# Differentiating littering, urban runoff and marine transport as sources of marine debris in coastal and estuarine environments

**DOI:** 10.1038/srep44479

**Published:** 2017-03-10

**Authors:** Kathryn Willis, Britta Denise Hardesty, Lorne Kriwoken, Chris Wilcox

**Affiliations:** 1 Geography and Spatial Sciences, School of Land and Food, University of Tasmania, Hobart, TAS, 7001, Australia; 2 Commonwealth Scientific and Industrial Research Organisation (CSIRO) Oceans and Atmosphere, Hobart, TAS 7000, Australia

## Abstract

Marine debris is a burgeoning global issue with economic, ecological and aesthetic impacts. While there are many studies now addressing this topic, the influence of urbanisation factors such as local population density, stormwater drains and roads on the distribution of coastal litter remains poorly understood. To address this knowledge gap, we carried out standardized surveys at 224 transect surveys at 67 sites in two estuaries and along the open coast in Tasmania, Australia. We explored the relative support for three hypotheses regarding the sources of the debris; direct deposition by beachgoers, transport from surrounding areas via storm water drains and coastal runoff, and onshore transport from the marine system. We found strong support for all three mechanisms, however, onshore transport from the marine reservoir was the most important mechanism. Overall, the three models together explained 45.8 percent of the variation in our observations. Our results also suggest that most debris released into the marine environment is deposited locally, which may be the answer to where all the missing plastic is in the ocean. Furthermore, local interventions are likely to be most effective in reducing land-based inputs into the ocean.

Marine debris is a critical problem to the safety and health of global marine environments. It is defined as any persistent, manufactured or processed solid material discarded, disposed of, or abandoned, in the marine and coastal environment^1^. Studies have shown that marine debris can affect the income of a region^2^, the safety of marine vessels^3^, survival of marine species^4^,^5^,^6^, and change the physical characteristics of the surrounding local marine environment^7^,^8^. The quantity of debris in the aquatic environment is predicted to continually increase due to a combination of poor disposal practices and rapidly increasing plastic production^9^.

Debris has been observed in a wide range of environments including estuaries^10^,^11^ and along beaches^12^,^13^,^14^. Estuaries are a key ecosystem for aquatic species as they provide habitat for spawning grounds and can act as sanctuaries for a range of species^15^,^16^. Estuaries within Australia are identified as threatened ecological communities^17^. In August 2003, marine debris was identified as a key threatening process under the Australian *Environment Protection and Biodiversity Conservation Act 1999 (EPBC Act*)^18^. However, relatively little research has been undertaken on shoreline debris found in Australian estuaries.

Most of the major cities in Australia are situated in coastal areas, within the catchment of an estuary. In 2011 it was estimated that 82% of Australians lived within 50 km of the coast^19^. The shores and catchments of these cities are heavily urbanised. Urbanisation alters waterways and overland flow through the installation of drains, roads and seawalls^20^,^21^. Household pollution and stormwater drains also contribute to shoreline debris^11^,^22^,^23^. However, terrestrial waterways are the main transport processes by which debris is transported from the land to the coast^24^,^25^,^26^. To date, no comparisons between coastal and estuarine debris have been presented in the scientific literature, and the relative importance of the various sources has not been explored empirically.

We carried out a scientifically rigorous survey and corresponding analysis comparing estuary and coastal shoreline debris. Our objective was to determine what are the anthropogenic factors associated with urbanisation that influence the distribution of estuary and coastal debris. We addressed the following questions:

- Do we find similar types and amounts of debris in coastal and estuarine sites?
- Does an increase in local population correlate with higher debris loads?
- Do we find higher debris loads where there is more infrastructure (e.g. stormwater drains, roads, car parks).
- What is the relative importance of direct deposition by coastal users, urban runoff and stormwater, and onshore deposition from the marine environment in the amount of debris found on shoreline environments?

## Results

**Figure 1:**
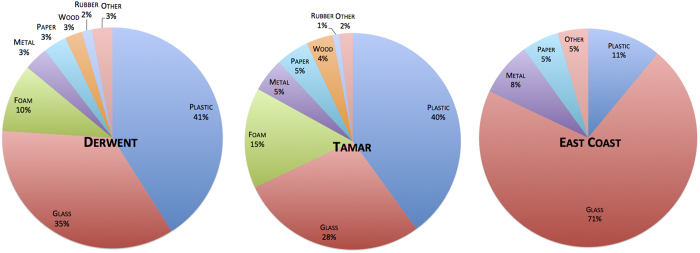
The percentage of different types of debris observed in the Derwent Estuary, Tamar Estuary and the east coast of Tasmania. Percentages are of total number of debris items observed in respective estuaries and coast.

A total of 78 transects were surveyed across 26 sites in the Derwent Estuary with an average of 3.0 transects per site. There were 93 transects surveyed across 29 sites in the Tamar Estuary with an average of 3.2 transects per site. Along the east coast 53 transects were surveyed across 12 sites with an average of 4.4 transects per site. A total 751 items of debris were observed in the Derwent, 308 items observed in the Tamar and 72 items observed along the east coast. Of the total debris counts in both estuaries, plastic items were the most abundant material type. Glass items were the next most observed followed by foam, metal, paper, wood and rubber (Fig. 1). Along the east coast glass was the most observed item, followed by plastic, metal and paper (Fig. 1).

### Estuaries

**Figure 2:**
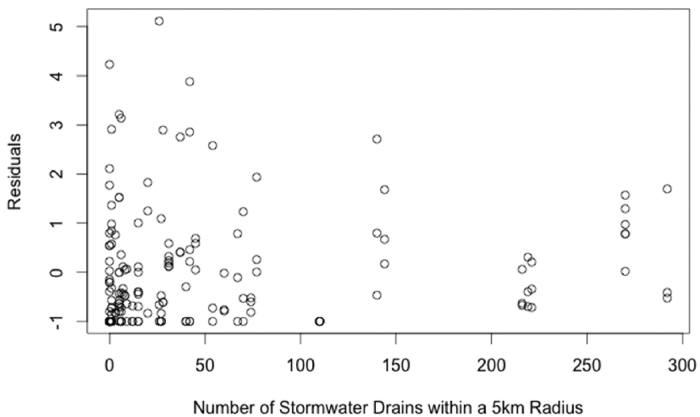
The effect of the number of stormwater drains within 5 km radius of each site has on total amount of debris observed, in the Derwent and Tamar Estuaries. Each point represents one transect.

**Figure 3:**
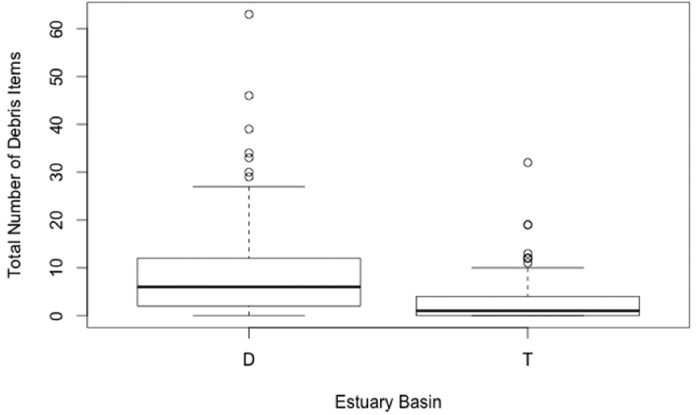
The effect of estuary basin on the total amount of debris observed. The Tamar Estuary has less debris than the Derwent Estuary. Along the x-axis, D = Derwent Estuary, T = Tamar Estuary.

A full model, including the terms distance downstream from uppermost sample, side of estuary, estuary basin, percentage of days wind blew onshore over the past 14 months at 1500 hours, stormwater drains within 5 km of a site, minimum distance from a site to a storm water drain outlet, distance to nearest public road, and population within 25 km of a site, captured 45.8% of the variation in the data (based on a deviance comparison, Tables 1 and 2). The model showed debris along an estuary shoreline increases significantly with an increase in the number of stormwater drains within 5 km radius of each site (Fig. 2). The east side of both estuaries had more debris than the west side and the upstream sampling sites had more debris than downstream sampling sites. Debris decreases along an estuary shoreline with an increase in distance from a site to a storm water drain outlet, i.e. the further the distance of the nearest stormwater drain to a site, the less debris will be observed. An increase in debris was found at sites closer to a public road and sites with a larger population within a 25 km radius. Overall, the Tamar Estuary had significantly less debris than the Derwent Estuary (Fig. 3).

### East Coast

**Figure 4:**
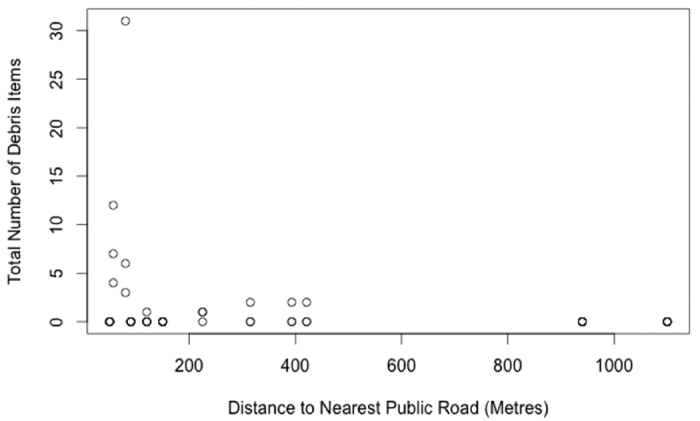
The effect the distance a site is from a public road has on the total amount of debris observed along the east coast of Tasmania. Each point represents one transect.

The full model, including the terms beach aspect, distance to nearest public road, water catchment population, percentage of days wind blew onshore over the past 14 months at 0900 and 1500 hours and if a beach clean-up had taken place in last 12 months, captured 91% of the variation in the data (based on a deviance comparison, Table 3 and 4). The full model (AIC = 89.31) showed that debris along Tasmania’s east coast decreases significantly the further a site is from a public road (Fig. 4). An increase in population within a water catchment showed a slight increase in the amount of debris present at a site; however, this relationship was not significant (Table 4).

### Importance of sources

The support, based on AIC, for the marine transport model was stronger than the support for either the beach user or stormwater drain model, although all three models were improvements in comparison with a null model (Tables 1 and 3). However, as the final full model had the lowest AIC score of all sub models, all sub models (i.e. different debris sources) contribute to explaining the variation in debris deposition along both estuaries and the east coast.

## Discussion

Overall, we observed less plastic than what has been reported in other similar studies^13^,^27^; 40% of all debris detected in the Derwent and Tamar estuaries was composed of plastic. This is markedly less than the 60–80% reported in other estuary debris studies^10^,^28^,^29^. Unexpectedly, in our coastal surveys, only 11% of total items were plastic. This was surprising given previous beach debris surveys in Tasmania found that plastic made up the largest proportion of debris items^30^,^31^. Furthermore, an Australia-wide survey found three-quarters of coastal debris observed was plastic^32^ and globally plastic typically represents 60–80% of debris observed on beaches^33^.

Glass made up the greatest proportion of debris on the east coast of Tasmania (71% of total items) and the high glass counts reduced the proportion of plastic we observed. As glass (typically found as fragments on our surveys, KW, pers. obs.) does not float, we presumed it arrived via local deposition. We suggest that the high proportions of glass observed on our surveys is likely due to the large number of beach users and visitors in the state. More visitors provide more opportunity for littering to occur. Furthermore, the areas surveyed are comprised of rocky substrate in many instances (50% of transects in the estuaries and 20% of transects along the east coast had a rocky substrate). Glass can readily shatter on the rocks and broken glass can become trapped in crevices, as we observed (KW pers. obs.).

Of the three transport sub-models, we found that the marine transport model was the best model predicting debris deposition in both estuaries and the east coast. Our estuary marine transport model shows that debris entering the estuary upstream becomes deposited on downstream shores by wind and currents. There is a westerly prevailing wind in Tasmania and we found a significant positive influence of onshore wind on debris deposition. These two factors can help explain why we found more debris on the eastern shores of both estuaries.

The inclusion of the stormwater drain and beach user terms in the best fitting overall model indicates both are contributing sources to debris deposition. Stormwater drains have been identified as a major contributor to shoreline debris in Australia^23^,^34^. Stormwater drains are an outflow point of litter in urban run-off. We found greater amounts of debris on shores with a greater number of stormwater drain outflows within a 5 km radius, even when we controlled for local population density. The greater number of stormwater drains, the higher probability that litter will exit a drain and be deposited on the shore.

Beach users have been shown to be main contributors to debris along coastal and estuarine shores^13^,^35^. The ease of access to a beach is a main factor to the number of beach visitors^36^. Beaches closer to roads are generally easier to access and have higher numbers of visitors. The estuary and east coast model included a positive distance to nearest public road. This suggests the shores closer to roads experienced more visitors to potentially deposit debris on the beach. We observed nearly three times the quantity of debris along the Derwent than the Tamar survey sites (Mean = 9.6 and 3.3 items per transect, respectively). The Derwent Estuary has a larger urban centre and double the population of the Tamar Estuary, which likely accounts for the difference observed.

Our study of the debris distribution on open coasts and urbanized estuaries provides an overall conceptual model for debris input and movement in coastal regions. Heavily urbanized upper reaches of estuaries have significant inputs of debris from both stormwater and beach users. These transport sources affect local estuarine sites, but also contribute to the pool of debris in the marine system. Wind, current, and wave transport then deposit debris, resulting in concentrated areas of debris in particular portions of the coastal margin. These concentrations are generally highest near the stormwater and beach user transport sources, and steadily decrease moving down the estuary and out onto the open coastline.

Jambeck *et al*.^9^ observed that plastic waste entering the marine system was one to three orders of magnitude greater than the reported mass of floating plastic in oceans globally and there is a long standing discussion about where the ‘missing’ plastic entering the oceans resides^34^,^37^. The marine transport of debris from upper estuary sources to local downstream shores observed can explain the disparity between land-based inputs and plastic in our oceans, suggesting that the majority of the material may be deposited in coastal regions close to its sources^9^,^38^.

Our research demonstrates that local solutions, such as improvements in infrastructure, public outreach (education) and rubbish bins at popular beaches are all likely to decrease the amount of debris on our shorelines^39^,^40^,^41^. Similarly, litter traps on stormwater drains have shown to significantly reduce debris entering the coastal margin. For example, within the Derwent Estuary, litter traps on stormwater drains capture 136 tonnes of litter per year^42^. Also, stormwater traps were shown to remove 44% of litter before it reaches the San Francisco Bay^43^.

In summary, local marine transport of debris from upstream sources to downstream shores was found to be the main driver of debris deposition. The types and amounts of debris on estuarine shores differed to what we found in the coastal zone. The difference in debris quantity was influenced by local population size and proximity to urban infrastructure. Shores with large local populations and a high number of stormwater drains had more debris. Additionally, we showed that easily accessible shorelines have more debris, due to their high frequency of beach visitors and subsequent increased chance of littering. These results emphasise the need and importance of targeting debris at the local level.

## Methods

### Survey area

**Figure 5:**
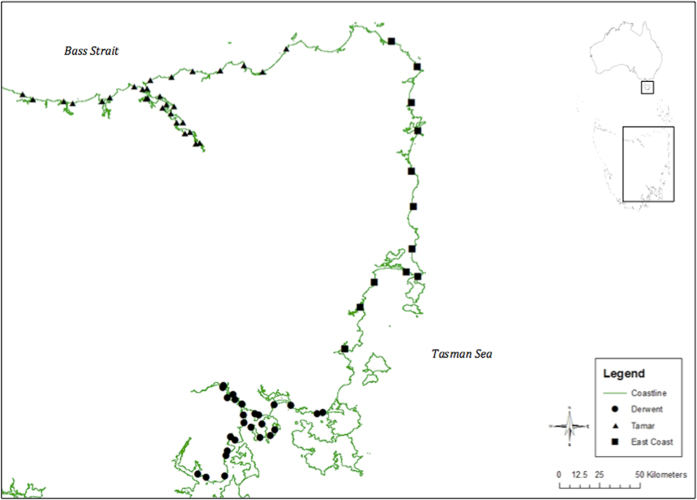
Survey sites in the Derwent Estuary, Tamar Estuary and east coast, Tasmania, Australia. Generated by Kathryn Willis using ArcGIS, [Desktop version 10.2.], (https://www.arcgis.com/features/index.html). LIST Coastline (MHWM) from theLIST ©State of Tasmania (Creative Commons Attribution 3.0 Australia License, https://creativecommons.org/licenses/by/3.0/au/legalcode).

Tasmania is an island south of the mainland of Australia, surrounded by the Indian Ocean to the west, Southern Ocean to the south and the Tasman Sea to the east. Tasmania’s prevailing winds are from the west. Three regions of Tasmania were selected for this research: the Derwent Estuary, the Tamar Estuary and the east coast of Tasmania (Fig. 5). Sampling was conducted between August 2014 and February 2015. Survey sites were distributed along each side of the estuaries, at 12.5 km intervals from a randomly selected starting location removing any bias in site selection. Where selected sites were inaccessible and could not be surveyed, the nearest site that could be accessed was surveyed.

### Transect surveys

Survey methodology was adapted from Hardesty *et al*.^32^. Transect surveys were conducted using stratified random sampling. A minimum of three and a maximum of six transects were conducted at each site. To avoid bias, such as the effect of higher traffic at the access point, the first transect commenced 50 m from the point of entry onto the beach. Subsequent transects were spaced a minimum of 50 m apart. When debris was found in the first three transects, the survey would terminate at the third transect. When no debris was found in the first three transects, additional transect surveys were carried out until debris was observed or a maximum of six transects had been surveyed. Each site was sampled once. To reduce any potential for inter-observer bias a single observer conducted all surveys.

Transects ran perpendicularly to the shoreline, starting at the water boundary and terminating two metres into the terrestrial coastal vegetation. The vegetation types ranged from coastal heathlands to dry eucalypt forests. Transects for swamp or marshland environments ran from the interface between the semi-submerged vegetation and open water, up to 2 m into vegetation on firm ground. All debris observed from a standing position was recorded according to material type and colour. Rubber was recorded separately from plastics, as it is frequently a natural product. To adhere with Hardesty *et al*.^32^ foam was also recorded separately from plastics. Seaweed, rocks and large drift wood items were not turned over when surveying for debris. Organic debris was not recorded unless it was identified as human modified (e.g. evidence of alteration by tools [building materials] or similar).

The following variables were recorded at each site: arrival time, number of engineered car parks, weather condition, direction of wind relative to the shore, wind speed, and number of people on the beach (Supplementary Information). The following variables were recorded at each transect: start and end time, transect length, distance from the start of the transect to the dominant debris line, beach gradient, substrate type of beach, colour of the substrate, physical structure of the backshore where the beach meets terrestrial vegetation, beach shape based on 25 m each side of the transect, and the aspect of the beach when facing the water (Supplementary Information). Only debris greater than 5.0 mm in length observed along each 2 m wide transect was recorded.

### Geographical Information System analysis

All Geographical Information System (GIS) analysis was performed on ArcGIS (version 10.2). GIS was used to calculate the shortest distance from a site to the nearest public road, the distance from each site to the nearest stormwater drain outlet, population within a 5 km and 25 km radius of each site and the population within the catchment of each site.

### Statistical analysis

The R statistical software^44^ was used for all statistical analysis. Generalised Additive Models, as implemented in the mgcv package in R^45^ were fitted to the estuarine and coastal data using a smooth spatial component based on a line feature to describe the location from the upper estuary shoreline outward to the open coastal shoreline. For the east coast a separate smooth spatial component using a line feature was used to describe the location from the north-eastern to the south-eastern coastline. In all cases the terms were initially included as smooth components, but then were reduced to parametric terms given their roughly linear estimates. We used a negative binomial distribution to model the count of debris items on our transects.

We tested hypotheses about the sources of debris at each site using three different sub models. The sub model for transport by surface runoff and stormwater drains included the following variables: number of cadastral parcels within a site’s catchment, number of storm water drain outlets in a 5 km radius of each site and the minimum distance from a site to a storm water drain outlet. The sub model for direct deposition by people included population within a 5 km and 25 km radius of the sites, number of people on the beach at the time of the survey, distance to the nearest public road, number of car parks at the access point. The sub model for deposition from the reservoir in the marine system included percentage of days wind blew onshore over the past 14 months at 0900 and 1500 hours, mean speed of onshore wind over the past 14 months, the aspect the shore faced, the estuary basin (Tamar or Derwent) and an interaction term for which the side of the estuary (east or west). The estuary basin and estuary side terms were excluded from the east coast models.

Environmental variables were also included that could affect deposition and re-suspension rates and detection of debris, including beach gradient, substrate type of beach, beach shape. Variables that could potentially introduce sampling error into data such as time since last clean-up, on-day wind speed and on-day weather were also included in our analyses.

Both transport models were run with every possible combination of terms. The Akaike Information Criterion (AIC) scores of each model were compared to determine the most parsimonious model. All terms from each parsimonious model were combined into a single aggregate model. The east coast data was analysed separately from the estuary data to account for the use of a different spatial component and to identify whether sources of debris differed between coastal and estuarine sites.

## Additional Information

**How to cite this article**: Willis, K. *et al*. Differentiating littering, urban runoff and marine transport as sources of marine debris in coastal and estuarine environments. *Sci. Rep.* **7**, 44479; doi: 10.1038/srep44479 (2017).

**Publisher's note:** Springer Nature remains neutral with regard to jurisdictional claims in published maps and institutional affiliations.

## Supplementary Material

Supplementary Information

## Figures and Tables

**Table 1:** AIC scores of term combinations for each hypothesis model for the Derwent and Tamar Estuaries.

Model	Number of Terms	Factors	AIC Score
Null	**0**		**993.91**
Stormwater	1	no. drain outlets in 5 km	966.12
	**2**	**no. drain outlets in 5 km, distance to nearest drain**	**961.04**
	3	no. drain outlets in 5 km, distance to nearest drain, population in water catchment	961.58
Beach Users	1	population in 25 km	965.54
	**2**	**population in 25 km, distance to public road**	**959.35**
	3	population in 25 km, distance to public road, population in 5 km	960.13
	4	population in 25 km, distance to public road, population in 5 km, cleanup	960.13
	5	population in 25 km, distance to public road, population in 5 km, cleanup, no. carparks	960.47
	6	population in 25 km, distance to public road, population in 5 km, cleanup, no. carparks, no. people on shore	962.30
Marine Transport	1	estuary basin	955.52
	2	estuary basin, aspect	953.12
	3	estuary basin, estuary side, distance	927.38
	**4**	**estuary basin, estuary side, distance, onshore wind 1500 hrs**	**922.68**
	5	estuary basin, estuary side, distance, onshore wind 1500 hrs, aspect	924.74
	6	estuary basin, estuary side, distance, onshore wind 1500 hrs, aspect, mean onshore wind speed	925.49
	7	estuary basin, estuary side, distance, onshore wind 1500 hrs, aspect, mean onshore wind speed, onshore wind 0900 hrs	927.38
	8	estuary basin, estuary side, distance, onshore wind 1500 hrs, aspect, mean onshore wind speed, onshore wind 0900 hrs, on-day wind speed	923.40
	9	estuary basin, estuary side, distance, onshore wind 1500 hrs, aspect, mean onshore wind speed, onshore wind 0900 hrs, on-day wind speed, on-day weather	923.95
Physical Characteristics	1	substrate	969.18
	2	substrate, gradient	967.69
	**3**	**substrate, gradient, shape**	**965.88**
Final Full Model	**11**	**distance, estuary side, estuary basin, onshore wind 1500 hrs, no. drain outlets in 5 km, distance to nearest drain, distance to public road, population in 25 km, shape, gradient, substrate**	**887.00**

Each line displays the best AIC score for each combination (i.e. the best score when a single term, two terms, three terms etc. are included in the model). The most parsimonious set of terms for each model are highlighted.

**Table 2:** Results of the GAM analysis of the Derwent and Tamar Estuaries final full model.

Parametric Coefficients	Estimate	Std. Error	z value	*p* value
Intercept	1.859E + 00	6.578E-01	2.827	0.0047
distance	−2.093E + 00	5.432E-01	−3.854	0.0001
estuary side	−1.257E + 00	3.124E-01	−4.024	5.73E-05
onshore wind 1500 hrs	1.745E + 00	6.760E-03	2.582	0.0098
estuary basin	−1.214E + 00	2.903E-01	−4.18	2.92E-05
no. drain outlets in 5 km	3.199E-03	1.401E-03	2.283	0.0224
distance to nearest drain	1.302E-05	2.479E-05	0.525	0.5994
distance to public road	2.202E-05	1.461E-04	0.151	0.8802
population in 25 km	−1.243E-07	2.972E-07	−0.418	0.6758
rock slab	−1.131E + 00	2.991E-01	−3.781	0.0002
sand	−7.706E-02	2.523E-01	−0.305	0.7600
marsh	−1.269E + 00	3.050E-01	−4.16	3.19E-05
pebble/gravel	1.297E + 00	2.985E-01	4.345	1.39E-05
gradient	−3.268E-01	1.657E-01	−1.973	0.0485
straight	2.357E-01	2.439E-01	0.966	0.3339
convex	2.036E-01	3.495E-01	0.583	0.5601
distance * estuary side	2.683E + 00	5.703E-01	4.705	2.54E-06

**Table 3:** AIC scores of term combinations for each hypothesis model for the east coast.

Model	Number of Terms	Factors	AIC Score
Null	**0**		**172.42**
Stormwater	**1**	**population in water catchment**	**150.95**
	2	population in water catchment, no. drain outlets in 5 km	158.37
	3	population in water catchment, no. drain outlets in 5 km, distance to nearest drain	151.82
Beach Users	1	distance to public road	143.08
	**2**	**distance to public road, cleanup**	**127.72**
	3	distance to public road, cleanup, population in 25 km	128.96
	4	distance to public road, cleanup, population in 25 km, no. carparks	130.88
	5	distance to public road, cleanup, population in 25 km, no. carparks, population in 5 km	131.75
	6	distance to public road, cleanup, population in 25 km, no. carparks, population in 5 km, no. people on shore	133.70
Marine Transport	1	mean onshore wind speed	147.93
	2	mean onshore wind speed, on-day wind speed	128.16
	3	mean onshore wind speed, on-day wind speed, distance	128.96
	**4**	**aspect, onshore wind 0900** **hrs, onshore wind 1500** **hrs, on-day wind speed**	**111.48**
	5	aspect, onshore wind 0900 hrs, onshore wind 1500 hrs, on-day wind speed, distance	113.46
	6	aspect, onshore wind 0900 hrs, onshore wind 1500 hrs, on-day wind speed, distance, mean onshore wind speed	114.76
Physical Characteristics	1	substrate	131.83
	2	substrate, gradient	113.99
	**3**	**substrate, gradient, shape**	**96.85**
Final Full Model	**10**	**population in water catchment, distance to public road, cleanup, aspect, onshore wind 0900** **hrs, onshore wind 1500** **hrs, on-day wind speed, substrate, shape, gradient**	**89.31**

Each line displays the best AIC score for each combination (i.e. the best score when a single term, two terms, three terms etc. are included in the model). The most parsimonious set of terms for each model are highlighted.

**Table 4:** Results of the GAM analysis of the east coast final full model.

Parametric Coefficients	Estimate	Std. Error	z value	*p* value
Intercept	3.699E + 01	2.678E + 01	1.381E + 00	0.167
population in water catchment	2.084E-01	1.506E-01	1.384E + 00	0.166
distance to public road	−5.492E-03	2.032E-03	−2.703E + 00	0.007
clean-up	−1.372E + 02	2.740E + 07	0.000E + 00	1.000
aspect	−9.072E-02	4.804E-02	−1.889E + 00	0.059
onshore wind 0900 hrs	−5.157E-01	2.540E-01	−2.030E + 00	0.430
onshore wind 1500 hrs	−1.602E-01	2.520E-01	−6.360E-01	0.525
wind speed	−5.252E-01	6.659E-01	−7.890E-01	0.430
sand	−1.309E + 00	6.766E-01	−1.934E + 00	0.339
rock slab	−5.755E-01	6.020E-01	−9.560E-01	0.053
gradient	−3.262E-01	4.933E-01	0.000E + 00	0.508
straight	2.343E-01	1.422E + 00	1.650E-01	0.869
convex	1.705E + 00	8.845E-01	1.927E + 00	0.054
