# Supplementary material for: Differentiating littering, urban runoff and marine transport as sources of marine debris in coastal and estuarine environments
Source: Sci Rep. 2017 Mar 10;7:44479. doi: 10.1038/srep44479 (PMC5345086; doi:10.1038/srep44479)
Supplement: Supplementary Information [file srep44479-s1.pdf]

Differentiating littering, urban runoff and marine transport as sources of marine debris in coastal and estuarine environments

Kathryn Willis, Britta Denise Hardesty, Lorne Kriwoken and Chris Wilcox

Description:
Survey data sheets for each site and each transect. The data sheets used in this study were provided by Dr. Britta Denise Hardesty.

**(Official use only)**	Beach number (unique identity code): ……………….	Survey Area (A, B, C, D, E, F, G): ……………….

SURVEY AREA CODE: **A** = Cape Tribulation – Bris; **B** = Bris – Melb; **C** = Melbourne – Streaky Bay; **D** = Streaky Bay – Perth; **E** = Perth – Broome; **F** = Broome – Darwin; **G** = Around Tasmania

MARINE DEBRIS BEACH SURVEY

Survey Guidelines:

- Complete one Beach survey form per site and one transect data form for each transect at the site. Record all coordinates in WGS84 datum only.
- Minimum of three transects and minimum of six per site.
  - Minimum of one transect located within each major habitat type (transects proportional to habitat type).
  - Transects located at least 50 m from beach access point (ideally not located both sides of access points, unless different habitat types).
  - Transects located at least 25 meters apart (ideally 50 meters).
  - Transect to include two meters into continuing backshore terrestrial vegetation.

**SURVEYOR DETAILS**		
Organisation:		*Organisation responsible for survey.*
Surveyor name:		*Name of chief surveyor.*
Contact number:		*Contact number for surveyor.*
Access point location:	Latitude: …………………………………………………………….<br>Longitude: ………………………………………………………..	*Latitude and longitude of access point where you enter the beach (dd.dddd).*
GPS accuracy:		*Accuracy (meters) of GPS at time of reading.*

**SITE DETAILS**		
State / Territory:		*State or territory in Australia beach is located.*
Beach name:		*Unique name of beach , if known.*
Survey date:		*Date survey undertaken (dd/mm/yyyy).*
Current weather:	Clear Rain/Storm Overcast Drizzle	*Circle best option to describe the weather.*
Wind speed:	0 1 2 3 4 5	*Circle Speed estimate:*<br>*0: calm (flat ocean)*<br>*1: light breeze (wavelets, <10km/h , <6 knots)*<br>*2: moderate breeze (small waves braking crests, 10-25km/h, 6-20 knots)*<br>*3: strong breeze (waves and many white caps, 25-49km/h, 21- 26 knots)*<br>*4: high wind (white caps and airborne spray, 50-65 km/h , 27-35 knots)*<br>*5: gale (high waves, foam and spray present, 65-85 km/h, 35-45 knots)*
Wind direction:<br>*(compass)*	N NE E SE S SW W NW N/A	*Direction from which wind is coming measured by the compass. N/A if no wind.*
Wind direction:<br>*(relative to shore)*	onshore offshore sideshore side-on side-off	*Onshore: wind blowing towards shore*<br>*Offshore: wind blowing towards sea*<br>*Sideshore: wind blowing parallel to shore*<br>*Side-onshore: wind blowing sideways and towards shore*<br>*Side-offshore: wind blowing sideways and towards sea*
Date of last clean up:		*If known.*
Number of humans:	Time of day (00:00): ……………………………………………..<br>Visible distance (m): ……………………………………………..<br>No. of people: ………………………………………………………	*Number of people counted in the visible area measured by instantaneous count. Visible distance is length of shore with a clear and unobstructed view.*
Comments:		*For example: entangled fauna, recent storms, shipwrecks, boat ramp in close proximity, coastal erosion or other conditions that may affect the survey.*

Transect Data

Beach Name:		Name of surveyor(s):	
Transect Number:		No. of surveyor(s):	
Transect width (m):		Transect Number _____ of _________	

Transect start:	Latitude: ……………………………………<br>Longitude: ……………………………………<br>GPS Accuracy: ……………………………………<br>Start Time (00:00): ……………………………………	*Latitude and longitude recorded in decimal degrees (dd.dddd).*<br>*Accuracy (in meters) of the GPS at time of reading.*<br>*Record Start Time of Transect*
Transect end:	Latitude: ……………………………………<br>Longitude: ……………………………………<br>GPS Accuracy: ……………………………………<br>End Time (00:00): ……………………………………	*Latitude and longitude recorded in decimal degrees (dd.dddd).*<br>*Accuracy (in meters) of the GPS at time of reading.*<br>*Record End Time of Transect*
Photo numbers:	Start of Transect: ……………………………………<br>End of Transect: ……………………………………	*Number of photo, taken from transect start and end point.*
Transect length (m):		*From waters edge to two meters into continual terrestrial vegetation (meters).*
Distance to dominant debris line (m):		*Distance from water edge to major debris line (in meters) at time of survey. Example 23 meters. If no obvious debris line use NA.*
Beach gradient:	1 2 3 4 5	*Difference in elevation from start to end of transect.*<br>*1 = < 1 m (less than hip height)*<br>*2 = 1-2 m (hip to head height)*<br>*3 = 2-4 m (1-2 body length)*<br>*4 = 4-8 m (2-4 body lengths)*<br>*5 = > 8 m (more than 4 body lengths)*
Substrate type:	Mud Sand Pebble / Gravel Boulders<br>Rock slab Mangrove	*Major substrate type.*
Substrate colour:	White / cream Yellow Orange Brown<br>Black Grey Red	*Predominant colour of substrate.*
Backshore type:	Cliff Seawall Urban building<br>Forest / Tree (> 3m) Shrub (< 3m) Dune<br>Grass - tussock Grass - pasture Mangrove	*Physical structure of backshore, where beach meets terrestrial vegetation.*
Beach exposure or shape:	Concave (cove) Straight Convex (headland)	*Shape of beach where survey is conducted. Based on 25m each side of transect.*
Aspect:	N NE E SE S SW W NW	*Direction when you are facing the water.*
Comments:<br>Stormwater Drain : Y / N : Dist to Transect : m<br>Rubbish Bin : Y / N : Dist to Transect : m		*For example: transect-related comments such as backshore flora, crossing paths, photo information, etc.*

Transect debris (type and colour): Record one mark (e.g. IIII) for each piece of rubbish larger than 1 cm^2 in size, within 1 metre each side of the transect line. If you find items other than those listed, add details to bottom of table.

**Rubbish Type**		**Colour of debris**									
		**Clear / translucent**	**White**	**Red/ pink**	**Orange**	**Yellow**	**Green**	**Blue / purple**	**Brown**	**Black**	**Grey / silver**
**Plastic**	Hard plastic										
	Plastic bags										
	Film-like plastics (glad wrap and chip bags)										
	Other soft plastics										
	Plastic packing straps										
	Net (estimate size)										
	Fishing line										
	Plastic (string, twine, rope)										
**Cloth**	Non-plastic (string, twine, rope)										
**Glass**	Glass										
**Metal**	Fish hook										
	Metal (hard)										
	Metal (soft, tinfoil)										
**Rubber**	Balloon										
	Other rubber items										
**Foam**	Polystyrene (foam, from esky's buoys etc.)										
	Other foam										
**Timber**	Wood (posts, beams, ship hulls)										
**Paper**	Cigarette butts										
	Paper										
**Other**											

Size classes: Sample debris type and size class at ten intervals along each transect.

**Sampling Interval**	**Distance from water (m)**	**Size Class**	**Type / colour**
1			
2			
3			
4			
5			
6			
7			
8			
9			
10			

1. Divide the total transect length by 10 to determine sampling interval, e.g. if transect is 35 m, interval = 3.5 m.

2. At each interval record the type and size of the first piece of rubbish encountered. If no rubbish is detected within the interval draw a line through the box and continue to next interval, e.g. if no rubbish is found within the second interval (3.5–7m), but six pieces were detected in the third interval (7–10.5m) mark a line in the box for sample 2, and record the size and type for only the first item detected in sample3
